# Dr. Nelson J. Henry Introduced in the Legislature a Bill Providing for the Organization of a State School of Public Health

**Published:** 1899-09

**Authors:** 


Dr. Nelson J. Henry, now a member of the N. Y. Assembly, recently introduced in the Legislature a bill providing for the organization of a State School of Public Health under the auspices of the University of the city of New York, and appropriating the sum of $25,000 for its establishment, and a like amount for its maintenance for the year beginning October 1, 1899. The bill has been favorably reported with an amendment by the Ways and Means Committee stipulating that the school thus incorporated shall furnish free instruction in sanitation to properly accredited members of boards of health of towns and villages, and also authorizing the school to conduct examinations regarding the purity of the water-supplies of the State.

